# The feasibility of 350 micron spatial resolution coronary MRA at 3T in humans

**DOI:** 10.1186/1532-429X-14-S1-O45

**Published:** 2012-02-01

**Authors:** Ahmed Gharib, Khaled Abd-Elmoniem, Vincent B Ho, Eszter Fodi, Daniel Herzka, Jacques Ohayon, Matthias Stuber, Roderic I Pettigrew

**Affiliations:** 1 Biomedical and Metabolic Imaging Branch, NIDDK, NIH, Bethesda, MD, USA; 2 NIBIB, NIH, Bethesda, MD, USA; 3 Johns Hopkins University, Baltimore, MD, USA; 4 Uniformed Services University of the Health Sciences, Bethesda, MD, USA

## Background

The purpose of this study was to (1) develop a high resolution 3T MRA technique with in-plane resolution approximate to that of MDCT and a voxel size of 0.35 × 0.35 × 1.5 mm3 and to (2) investigate the image quality of this technique in healthy subjects and preliminarily in patients with known coronary artery disease (CAD).

## Methods

A 3T coronary MRA technique optimized for an image acquisition voxel as small as 0.35 x 0.35 x 1.5mm3 (HRC) was implemented and the coronary arteries of twenty two subjects were imaged. These included 11 healthy subjects (average age 28.5 years old, five males) and 11 subjects (average age 52.9 years old, five females) with CAD as identified on multidetector coronary computed tomography (MDCT). Additionally, the 11 healthy subjects were imaged using a method with a more common spatial resolution of 0.7×1×3 mm3 (RRC). Qualitative and quantitative comparisons were made between the two MRA techniques.

## Results

Normal vessels and CAD lesions were successfully depicted at 350x350µm2 in-plane resolution with adequate signal-to-noise ratio (SNR) and contrast-to-noise ratio (CNR). The CAD findings were consistent among MDCT and HRC. The HRC showed a 47% improvement in sharpness despite a reduction in SNR (reduced by 72%) and CNR (reduced by 86%) compared to the RRC.

## Conclusions

This study, as a first step towards substantial improvement in the resolution of coronary MRA, demonstrates the feasibility of obtaining at 3T a spatial resolution that approximates that of MDCT. The acquisition in-plane pixel dimensions are as small as 350µm x 350µm with a 1.5 mm slice thickness. While SNR is lower, the images have improved sharpness resulting in image quality that allowed qualitative identification of disease sites on MRA consistent with MDCT.

## Funding

NIH.

**Figure 1:**
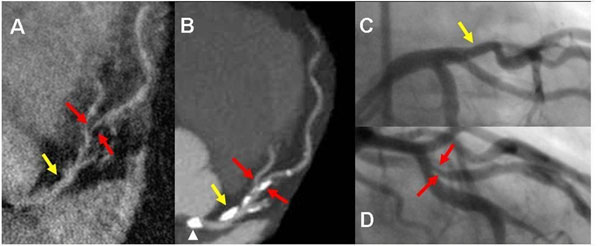
Demonstrates none-significant stenosis in the proximal LAD (yellow arrow) and significant stenosis in mid LAD (red arrows) in a 76 year old male seen both on HRC (A) 320-detectors CT (B), and conventional angiogram (C and D). Despite compromised flow in high grade stenosis, signal is adequate for visualization on the HRC image. A. Multiplanar reformatted image of HRC MRA. B. Maximum intensity projection (MIP) in left anterior view of the LM/LAD. C. and D. Conventional angiogram of LAD in left anterior oblique views. Note calcification (arrow head) in the left cusp partially projecting over the origin of the LM on the MIP due to image plane (B) is not causing severe narrowing as seen on the conventional angiogram (C) or HRC (A).

